# Prevalence and Genetic Characterization of Carbapenem- and Polymyxin-Resistant *Acinetobacter baumannii* Isolated from a Tertiary Hospital in Terengganu, Malaysia

**DOI:** 10.1155/2014/953417

**Published:** 2014-03-19

**Authors:** Soo-Sum Lean, Zarizal Suhaili, Salwani Ismail, Nor Iza A. Rahman, Norlela Othman, Fatimah Haslina Abdullah, Zakaria Jusoh, Chew Chieng Yeo, Kwai-Lin Thong

**Affiliations:** ^1^Institute of Biological Sciences, Faculty of Science, University of Malaya, 50603 Kuala Lumpur, Malaysia; ^2^Faculty of Agriculture, Biotechnology and Food Sciences, Universiti Sultan Zainal Abidin, 20400 Kuala Terengganu, Malaysia; ^3^Faculty of Medicine and Health Sciences, Universiti Sultan Zainal Abidin, 20400 Kuala Terengganu, Malaysia; ^4^Department of Pathology, Hospital Sultanah Nur Zahirah, 20400 Kuala Terengganu, Malaysia

## Abstract

Nosocomial infection caused by *Acinetobacter baumannii* is of great concern due to its increasing resistance to most antimicrobials. In this study, 54 nonrepeat isolates of *A. baumannii* from the main tertiary hospital in Terengganu, Malaysia, were analyzed for their antibiograms and genotypes. Out of the 54 isolates, 39 (72.2%) were multidrug resistant (MDR) and resistant to carbapenems whereas 14 (25.9%) were categorized as extensive drug resistant (XDR) with additional resistance to polymyxin B, the drug of “last resort.” Pulsed-field gel electrophoresis analyses showed that the polymyxin-resistant isolates were genetically diverse while the carbapenem-resistant isolates were clonally related. The 14 XDR isolates were further investigated for mutations in genes known to mediate polymyxin resistance, namely, *pmrCAB*, and the lipopolysaccharide biosynthesis genes, *lpxA*, *lpxC*, *lpxD,* and *lpsB*. All 14 isolates had a P102H mutation in *pmrA* with no mutation detected in *pmrC* and *pmrB*. No mutation was detected in *lpxA* but each polymyxin-resistant isolate had 2–4 amino acid substitutions in *lpxD* and 1-2 substitutions in *lpxC*. Eight resistant isolates also displayed a unique H181Y mutation in *lpsB*. The extent of polymyxin resistance is of concern and the novel mutations discovered here warrant further investigations.

## 1. Introduction

*Acinetobacter baumannii* is a Gram-negative bacterium increasingly found in the hospital environment due to its ability to survive for long periods on inanimate objects [1, 2]. Nosocomial* A. baumannii* isolates are mostly multidrug resistant (MDR) (i.e., resistant towards more than three classes of antibiotics) [3]. However, extensive drug resistant (XDR) isolates, which are resistant to all but one or two classes of antibiotics, and even pandrug resistant (PDR) isolates that are resistant to all classes of antibiotics, are developing at an alarming rate [4]. Since* A. baumannii* has the ability to colonize both viable and damaged tissues and is also resistant towards nearly all antimicrobials, it has become a cause of great concern. Carbapenems are among the very few antibiotics left that can be used for the treatment of* A. baumannii* infections. Nevertheless, the efficacy of carbapenems is increasingly compromised by the rapid emergence of carbapenem-hydrolysing *β*-lactamase enzymes. Several types of class D *β*-lactamases including OXA-23, OXA-24, OXA-58, and intrinsic OXA-51-like enzymes are known to be important contributors to carbapenem resistance [5].

The rapid development of carbapenem-resistant MDR* A. baumannii* has led to the use of polymyxins (in particular polymyxin B and colistin or polymyxin E) as the drug of “last resort” [6]. Polymyxins are cyclic, positively charged peptide antibiotics capable of posing antimicrobial activities to a broad variety of Gram-negative pathogens, including* A. baumannii,* due to their interaction with the lipid A moiety of lipopolysaccharide (LPS). This leads to the disorganization and disruption of the outer membrane integrity, causing cytoplasmic leakage [7]. Unfortunately, the intensive use of the polymyxins in recent years has led to the emergence of polymyxin heteroresistant and resistant* A. baumannii*, as high as 40.7% reported in Spain [8] and 30.6% in Korea [9].

The basis of polymyxin resistance in* A. baumannii* has only recently been investigated and several mechanisms have been proposed. Several genetic loci have been implicated in the resistance towards polymyxins in* Acinetobacter*, namely, the* pmrCAB* operon [10, 11] and the* lpxA*,* lpxC*,* lpxD* [12, 13], and* lpsB *genes, that are involved in LPS biosynthesis [14]. Resistance can arise through mutations in the two-component system PmrAB, in which the downstream target PmrC catalyzes the addition of phosphoethanolamine to the lipid A component of LPS [10, 11, 15]. This modification reduces the net negative charge of the outer membrane thus reducing the affinity of polymyxins for the target. Park et al. (2011) [16] also showed that increased expression of* pmrAB* could be essential for colistin resistance in* A. baumannii*. Mutations or insertions in the genes encoding the lipid A biosynthesis machinery, namely, the* lpxA*,* lpxC*, or* lpxD* genes, also mediate polymyxin resistance by abolishing the production of LPS, thereby eliminating the target of polymyxins [12, 13, 17]. Hood et al. (2013) [14] recently reported the identification of over 20 genes that are implicated in inducible colistin tolerance in* A. baumannii*, with majority of the genes involved in processes that protect the bacterium from osmotic stress. One of the genes identified,* lpsB*, encodes a glycosyltransferase involved in LPS biosynthesis. Polymyxin resistance in* A. baumannii* thus developed through intrinsic mechanisms that respond and adapt to polymyxin treatment and not through acquisition of horizontally transferred resistance genes [12, 13].

To our knowledge, there has been no published data on polymyxin-resistant isolates of* A. baumannii* from Malaysia. In this study, we determined the resistance profiles of* A. baumannii* isolated from Hospital Sultanah Nur Zahirah (HSNZ), the main tertiary hospital located in the state of Terengganu in the east coast of Peninsular Malaysia. The genetic diversity of the* A. baumannii* isolates was determined by pulsed-field gel electrophoresis (PFGE). This study also aims to determine the presence and distribution of genes encoding the class D carbapenemase *bla*_OXA-51_, *bla*_OXA-23_, *bla*_OXA-24_, and *bla*_OXA-58_, as well as the class B carbapenemase (or metallo-*β*-lactamase) genes *bla*_VIM_ and *bla*_IMP_ in the clinical isolates of* A. baumannii*. Besides that, we also aim to investigate the type of mutations that occur within the* pmrCAB*,* lpxA*,* lpxC*,* lpxD,* and* lpsB* genes that may confer resistance to polymyxin. Findings from this research project will help to determine the antimicrobial resistance, distribution of carbapenem and polymyxin resistance determinants, and the clonality of the* A. baumannii* isolates from HSNZ, thereby providing valuable information regarding this important nosocomial pathogen in this region.

## 2. Materials and Methods

### 2.1. Bacterial Isolates: Identification and Genotyping

Fifty-four nonrepeat clinical isolates of* A. baumannii* were collected throughout 2011 from sporadic cases of infection in Hospital Sultanah Nur Zahirah (HSNZ). The isolates were isolated and initially identified by standard biochemical methods at the clinical microbiology laboratory. Single colonies of* A. baumannii* were then cultured into nutrient broth and incubated at 37°C for 24 hours. Confirmation of the 54 isolates was done by 16S rDNA amplification and sequencing. Genomic DNA was extracted from the bacterial isolates using the Wizard Genomic DNA Purification Kit (Promega, Madison, WI, USA) and subjected to PCR using 16S rDNA universal primers [18] (Table 1). Strain typing of* A. baumannii* was carried out by pulsed-field gel electrophoresis (PFGE), as previously described [19]. Briefly, chromosomal DNA for PFGE analysis was prepared in agarose gel blocks, digested with restriction endonuclease* Apa*I (Promega, Madison, WI, USA). Restriction fragments were separated by using the CHEF Mapper (Bio-Rad, Hercules, CA, USA) with 0.5 × TBE buffer for 26 h at 14°C with pulse times of 2–40 s.* Xba*I-digested* Salmonella enterica* ser. Braenderup H9812 was used as the molecular size standard. The PFGE profiles were analyzed using BioNumerics version 6.0 software (Applied Maths, Kortrijk, Belgium). The unweighted pair group method with averages (UPGMA), with a position tolerance for comparison of 1.5%, was used to produce the dendrogram.

### 2.2. Antimicrobial Susceptibility: MIC Determination for Carbapenems and Polymyxin B

The Kirby-Bauer disc diffusion method was used to test the susceptibility of the* A. baumannii* isolates to the following 12 antibiotics (Oxoid Ltd., Basingstoke, UK): gentamicin (10 *μ*g), tobramycin (10 *μ*g), amikacin (30 *μ*g), ciprofloxacin (5 *μ*g), levofloxacin (5 *μ*g), piperacillin-tazobactam (100/10 *μ*g), cefotaxime (30 *μ*g), ceftazidime (30 *μ*g), cefepime (30 *μ*g), ampicillin-sulbactam (10/10 *μ*g), tetracycline (30 *μ*g), and doxycycline (30 *μ*g). The antibiotic susceptibility test was carried out on Mueller Hinton (Hi media) Agar swabbed with 0.5 McFarland standard suspension of* A. baumannii* and incubated at 37°C for 24 h. Guidelines from the Clinical and Laboratory Standard Institute (CLSI) [20] were used to interpret the diameters of the inhibition zones produced.

Bacterial cultures of* A. baumannii* adjusted to 0.5 McFarland standard were used to determine the minimal inhibitory concentration (MIC) to imipenem and meropenem using M.I.C. Evaluator (M.I.C.E.) strips (Oxoid Ltd., Basingstoke, UK). Inoculated media were incubated at 35 ± 2°C for 16–20 h. The MIC values of the respective inhibition ellipses that interact with the strips were taken and recorded. Data were analyzed according to the guidelines given for M.I.C.E. strips and the MIC was determined.

MIC values for polymyxin B were determined by the agar dilution method [21]. Agar dilution plates were prepared by using Mueller-Hinton Agar added with concentrations of 2, 4, 8, 16, 32, 64, and 128 *μ*g/mL polymyxin B sulfate (Sigma-Aldrich, St. Louis, MO, USA). A multipoint inoculator was used to deliver bacterial suspensions adjusted to 0.5 McFarland standard onto the agar surface and the agar subsequently incubated at 35 ± 2°C for 16–20 h. The MIC value for polymyxin B was determined as the lowest concentration of the antibiotic in which the bacteria were susceptible. The standard breakpoint for resistance according to the CLSI guideline [20] was ≥ 4 *μ*g/mL.

### 2.3. Detection of Carbapenem- and Polymyxin-Resistant Determinants, DNA Sequencing, and Sequence Analysis

The PCR primers and conditions used to detect the presence of several carbapenem resistance genes such as *bla*_OXA-23_, *bla*_OXA-51_, *bla*_OXA-24_, *bla*_OXA-58_, *bla*_IMP_, and *bla*_VIM_ are indicated in Table 1. The validity of the amplicons was determined by DNA sequencing. Briefly, the amplified products were purified by using the Wizard SV Gel and PCR Clean-Up System (Promega, Madison, WI, USA) and then submitted to a commercial facility (First Base Sdn. Bhd., Malaysia) for sequencing.

Genetic determinants that have been implicated in conferring polymyxin resistance in* A. baumannii*, namely, the* pmrCAB*,* lpxA*,* lpxC*,* lpxD,* and* lpsB* genes, were PCR-amplified using specific primers and conditions as shown in Table 1. Purified PCR products were then sequenced at the commercial facility (First Base Sdn. Bhd., Malaysia).

DNA sequences obtained were edited using BioEdit v7.0.9 (downloadable from http://www.mbio.ncsu.edu/bioedit/page2.html) and analyzed with BLAST at the National Center of Biotechnology Information website (http://www.ncbi.nlm.nih.gov/BLAST). Sequence analysis was also done by using ExPASy translate tool at the Swiss Institute of Bioinformatics website (http://web.expasy.org/translate/) and ClustalW2 multiple sequence alignment program (http://www.ebi.ac.uk/Tools/msa/clustalw2/).

## 3. Results

### 3.1. Antibiotic Susceptibility Profiles of* A. baumannii*

DNA sequence analyses of the 16S rRNA amplicons showed ≥99% sequence identity with* A. baumannii* 16S rRNA genes validating that all 54 isolates used in this study were* A. baumannii*.

High percentages of antibiotic resistance were observed for the following antibiotics: tetracycline (87%), piperacillin-tazobactam (72.2%), cefotaxime (72.2%), ceftazidime (72.2%), cefepime (72.2%), levofloxacin (70.4%), ampicillin-sulbactam (68.5%), gentamicin (66.7%), ciprofloxacin (66.7%), tobramycin (64.8%), doxycycline (61.1%), and amikacin (57.4%). The isolates also showed high resistance for carbapenems with 77.8% of the isolates resistant to meropenem and 74.1% resistant to imipenem. The majority of the carbapenem-resistant isolates showed MIC values of >32 *μ*g/mL. Of the 54 isolates, 39 (or 72.2%) could be categorized as multidrug resistant (MDR)—that is, resistant to more than three classes of antibiotics. All MDR isolates were resistant to meropenem and imipenem. Polymyxin resistance was assessed by determining the MIC values for polymyxin B. Of the 54 isolates, 14 (or 25.9%) were categorized as polymyxin resistant (MIC ≥ 4 *μ*g/mL). Four of these isolates had MIC values of >128 *μ*g/mL for polymyxin B. All 14 polymyxin-resistant isolates could also be categorized as extensive drug resistant (XDR) isolates based on the criteria proposed by Magiorakos et al. (2012) [22].

### 3.2. Detection of Class D and Class B Carbapenemase Genes

PCR was used to detect the presence of the class D carbapenemase genes *bla*_OXA-51_, *bla*_OXA-23_, *bla*_OXA-24_, and *bla*_OXA-58_. All 54* A. baumannii* isolates yielded *bla*_OXA-51_ amplicons of the expected size of 353 bp. Sequence analyses of the amplified products confirmed their identity as *bla*_OXA-51_ or *bla*_OXA-51-like_ gene. Forty-one (75.9%) of the 54 isolates yielded *bla*_OXA-23_ amplicons of the expected size of 501 bp, and sequencing of the amplified products validated the identity as *bla*_OXA-23_. None of the isolates harbor the *bla*_OXA-24_ or the *bla*_OXA-58_ genes. Similarly, no class B metallo-*β*-lactamase (MBL) genes, *bla*_IMP_ or *bla*_VIM_, were found in all the isolates tested.

### 3.3. Sequence Analyses of Polymyxin Resistance Determinants

All 14 polymyxin B resistant isolates were each subjected to PCR amplifications that target the* pmrCAB*,* lpxA, lpxC*,* lpxD,* and* lpsB* genes and the resulting amplicons were sequenced. The three isolates (AC6, AC11, and AC19) that were susceptible to polymyxin B were used as controls.

In this study, we found as many as 11 different point mutations within the* pmrB* gene when compared to the reference isolates ATCC 17978 and ATCC 19606 but these mutations were similarly found in the polymyxin-sensitive isolates. Thus, when the* pmrB *sequences of 14 polymyxin-resistant isolates were compared with the 3 polymyxin-sensitive isolates, no mutations within the* pmrB* gene could be found. Similarly, when comparing the sequences of the response regulator gene* pmrA*, 3 different point mutations (R149K, T153I, and Q165P) were observed but these mutations were commonly found in both sensitive and resistant isolates. However, all 14 resistant isolates had a P102H mutation within* pmrA* that was absent in the sensitive isolates (Table 2). When comparing the sequences for the* pmrC* gene that encodes lipid A phosphoethanolamine transferase, up to 8 point mutations were found in comparison with the reference isolates. However no difference was found amongst the polymyxin-sensitive and polymyxin-resistant isolates.

The* lpxA*,* lpxC,* and* lpxD* genes encode the first three enzymes in the lipid A biosynthesis pathway. No mutation was found in* lpxA*. In contrast,* lpxD* showed up to 4 amino acid mutations in each resistant isolate while* lpxC* had 1-2 amino acid mutations (Table 2). The 14 resistant isolates had the K141R, S158R, or both mutations in* lpxC* which encodes the enzyme involved in the second step of the lipid A biosynthesis pathway. The* lpxD* gene showed the most number of mutations variation among the resistant isolates with a total of 13 different amino acid substitutions. Each resistant isolate had between 2 and 4 amino acid substitutions in* lpxD* and most of the mutations occurred between amino acid residues 150 and 190 of LpxD. Further comparison of the* lpxD* sequence was carried out with known polymyxin-sensitive isolates in the database and results of the multiple sequence alignment (data not shown) indicated that the amino acid substitutions found in our resistant isolates are unique.

The* lpsB* gene encodes a glycosyltransferase involved in the biosynthesis of the LPS core and was recently implicated in* A. baumannii *colistin resistance [14]. Comparison of* lpsB* sequences among the 14 resistant isolates, the 3 sensitive isolates, and the 2 ATCC reference isolates initially showed that each resistant isolate contained as many as 4 amino acid substitutions in* lpsB*. However, when the* lpsB* sequence from other polymyxin-sensitive isolates in the database was taken into account, the majority of these amino acid substitutions were found to be common among the other polymyxin-sensitive isolates. The only unique mutation was H181Y and this substitution was found in 8 of the 14 resistant isolates (Table 2). The remaining 6 resistant isolates did not harbor any unique mutation within* lpsB*.

### 3.4. Genotyping of* Acinetobacter baumannii* Isolates by Pulsed-Field Gel Electrophoresis

**Figure 1:**
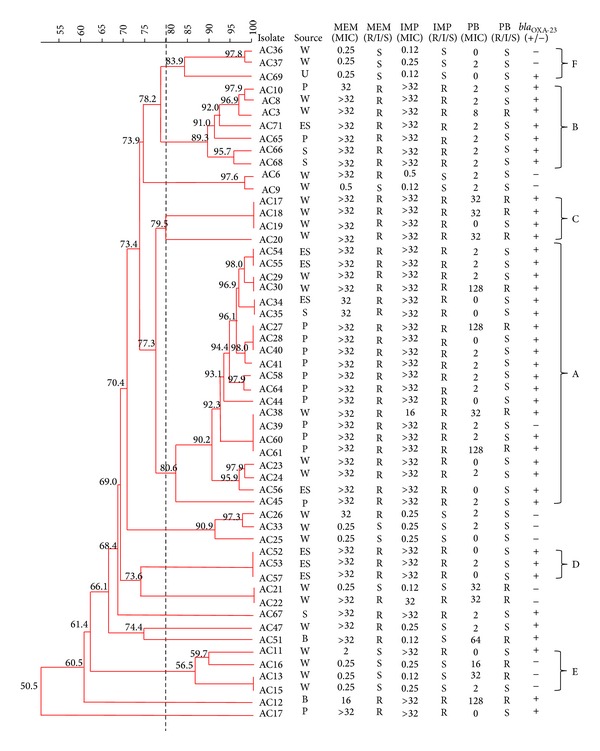
Dendrogram of the 54* A. baumannii* isolates from Hospital Sultanah Nur Zahirah (HSNZ) using unweighted pair group arithmetic means methods (UPGMA) from* Apa*I PFGE profiles. The dotted vertical line indicates the cutoff point of 80% similarity. The sources of isolation for the* A. baumannii* isolates are as indicated (wound, W; pus, P; endotracheal secretion, ES; skin, S; urine, U; blood, B). The MIC values for imipenem (IMP), meropenem (MEM), and polymyxin B (PB) for the isolates are shown as are their susceptibility states (resistant, R; intermediate, I; susceptible, S) according to CLSI (2012) [20]. PCR detection results for *bla*_OXA-23_ are also indicated (“+” for present; “−” for absent). The different clusters at 80% similarity are arbitrarily designated (A)–(E).

PFGE analysis of the 54* Apa*I-digested* A. baumannii* gave 15 reproducible profiles (pulsotypes) with a Dice coefficient, *F*, ranging from 0.90 to 1.00. Cluster analysis of the* Apa*I pulsotypes grouped the 54* A. baumannii* isolates into 9 clusters with a cutoff point at 80% similarity (Figure 1). Carbapenem-resistant isolates were grouped into four main clusters, A–D, while carbapenem-susceptible isolates were grouped into two clusters, E and F.

Cluster A is the largest cluster with 21 carbapenem-resistant isolates which were closely related (81.6% similarity), with 4 band differences at most (*F* value = 0.90–1.00). Four of the isolates within the A cluster were also polymyxin resistant and XDR. In addition, all the carbapenem-resistant isolates in clusters A–D were all positive for *bla*_OXA-51-like_ and *bla*_OXA-23_, whereas the susceptible isolates in clusters E and F were negative for *bla*_OXA-23_. However, there was no clustering among pulsotypes of the polymyxin-resistant, XDR isolates. In fact, isolates with identical PFGE patterns would differ in their resistance to polymyxin B. For the three isolates in cluster C which shared identical PFGE patterns, two of these (AC17 and AC18) were resistant to polymyxin B but the third isolate, AC19, was polymyxin sensitive. Both AC17 and AC18 shared identical mutations within the polymyxin-resistance determinants (i.e., E50D and V141I in* lpxD*, K141R in* lpxC,* and H181Y in* lpsB*). Likewise, AC29 and AC30 within cluster A shared an identical PFGE pattern but AC29 was sensitive to polymyxin B whereas AC30 was resistant.

## 4. Discussions

This study showed that the HSNZ isolates of* A. baumannii* were highly resistant to most antibiotics. Out of 54 isolates, 47 (87%) were resistant to tetracycline, which was the highest resistance among the 12 antibiotics tested. The percentages of resistance for carbapenems were also high with 77.8% for meropenem and 74.1% for imipenem. Although the percentage of carbapenem resistance was lower when compared to the rates reported from another medical facility in Kuala Lumpur (96.5% for imipenem and 98.2% for meropenem [19]), the finding that more than 50% of the isolates from HSNZ were carbapenem resistant is certainly a cause for concern as carbapenems have been the drugs of choice for* Acinetobacter* infections for over a decade [23, 24].

The overuse of carbapenems in hospitals to treat* Acinetobacter* infections has led to outbreaks of carbapenem-resistant* A. baumannii* [25]. Outbreaks of isolates carrying genes encoding OXA-type carbapenemase have increasingly been reported worldwide in recent years [18]. There are eight subgroups of OXA carbapenemases, amongst which are the OXA-23-like, OXA-24-like, OXA-51-like, and OXA-58-like groups [26]. The *bla*_OXA-51-like_ gene was found in all 54* A. baumannii* isolates and this was to be expected as the gene is naturally occurring in* A. baumannii* where it may be overexpressed leading to carbapenem resistance [23]. Overexpression of *bla*_OXA-51_ usually occurs due to the insertion of the IS*Aba1* insertion sequence upstream of the gene which provides the strong promoter sequence [23]. Forty-one out of the 54 isolates of* A. baumannii* in this study were positive for the *bla*_OXA-23_ gene. Of these 41 isolates, 37 were resistant to both meropenem and imipenem.

The second and third subgroups of carbapenem hydrolyzing *β*-lactamases were the OXA-24-like enzymes and OXA-58-like enzymes [23] encoded by *bla*_OXA-24_ and *bla*_OXA-58_ genes, respectively. In this study, none of the 54 isolates were found to harbor the *bla*_OXA-24_ and *bla*_OXA-58_ genes, similar to recent findings from Thailand [27]. Likewise, none of the isolates carried the metallo-*β*-lactamase genes, *bla*_IMP_ and *bla*_VIM_, both of which have not been reported in* A. baumannii* in this region. However, reports from Korea [28, 29] showed that these two genes were implicated in carbapenem resistance.

The recent emergence of polymyxin-resistant and heteroresistant* A. baumannii* [6] has necessitated the need to understand the mechanism of the development of polymyxin resistance in this pathogen. Since this older drug has recently returned in use and has been noted as the drug of last resort for* Acinetobacter* infections [6], we investigated the antibiotic resistance of the HSNZ isolates towards polymyxin B. It was found that 14 of the 54 isolates (or 25.9%) were resistant to polymyxin B, of which 4 had MIC values for polymyxin B >128 *μ*g/mL. The mechanism for polymyxin resistance in* A. baumannii* has only recently been investigated and the main mechanism appeared to be either covalent modification of the lipid A portion of LPS [11, 15] or disruption of LPS biosynthesis [13, 16]. By modification and/or mutations in the amino acid sequences, negative charges on the outer membrane can be reduced, leading to reduction in the affinity of the positively charged polymyxin component, hence giving rise to polymyxin resistance [10, 11, 15].

It is interesting that the 14 polymyxin-resistant isolates displayed an identical P102H mutation within the* pmrA* gene and this mutation was identical to that previously reported by Adams et al. (2009) [10] in a colistin-resistant derivative of* A. baumannii* AB0057. Overexpression of* pmrAB* and mutations within these genes, especially* pmrB*, were reported to contribute to polymyxin resistance [10, 11, 16]. As expression levels of* pmrAB* were not determined in this study and no mutation within* pmrB* was found, we are not certain if the P102H mutation in* pmrA* is a contributing factor to the polymyxin resistance in the 14 XDR isolates. However, analysis of the LPS biosynthesis genes identified novel mutations especially in the* lpxD*,* lpxC,* and* lpsB* genes. All 14 XDR isolates had mutations in both* lpxD* and* lpxC* genes whereas 8 out of the 14 isolates showed an identical H181Y substitution in* lpsB*. It does appear that disruption in the LPS biosynthesis genes could be associated with polymyxin resistance in the* A. baumannii* isolates studied. The effects of these mutations on the LPS of these isolates need to be further investigated as they differ from the mutations previously reported [30]. Furthermore the crystal structure of the* A. baumannii* LpxD protein was recently elucidated [31] and one of the mutations (G186S) lies within the proposed active site of the enzyme. Nevertheless, it should be noted that in a recent study [14], screening of transposon mutant libraries led to the identification of more than 20 genes that may be involved in inducible colistin resistance in* A. baumannii*. Most of these genes converge on pathways involved in osmotolerance, cell envelope biosynthesis along with protein folding [14]. The role that these factors may play in the development of polymyxin resistance among the 14 XDR isolates would also need to be investigated.

PFGE results indicated that the polymyxin-resistant isolates were genetically diverse. This is in contrast to the carbapenem-resistant isolates which were clonally related. The carbapenem-resistant cluster A was the largest cluster inferring that this is the major clonal group of* A. baumannii* existing within the hospital in 2011. Four of the 21 isolates within the A cluster were polymyxin resistant and XDR with three of these isolates having MIC values for polymyxin B >128 *μ*g/mL. Similar results were obtained whereby polymyxin-resistant isolates were found in clusters E, B, and C although the MIC values for these isolates were ≤32 *μ*g/mL. The clustering of carbapenem-resistant isolates has indeed been previously reported [32, 33]. Carbapenem resistance is due mainly to the acquisition of carbapenemases such as OXA-23 which are encoded on plasmids and other mobile elements. Isolates that acquire such genes will then undergo clonal expansion [34, 35]. In contrast, polymyxin resistance has been shown to develop through mutations in certain determinants in response to selection pressure [10–13, 17]. Thus these isolates were likely to develop randomly in the presence of polymyxins. Since polymyxin resistance is due to alterations or the complete absence of the outer membrane of LPS, it may incur a fitness cost to the bacterium. Therefore these isolates may not persist in the absence of polymyxin which could explain the random, nonclustering nature of the polymyxin-resistant isolates. This was indeed reported in a recent paper [36] where colistin-susceptible isolates outcompeted resistant isolates upon withdrawal of colistin in three out of four cases. However, in one case, an isolate with a* pmrB* L271R mutation appeared to be fit enough to be transmitted in the absence of colistin and not be outcompeted by susceptible isolates [36]. Colistin-resistant* A. baumannii* has also been reported to have reduced virulence in mice [37] although a more recent report indicated contrasting results whereby colistin resistance may not necessarily intrinsically affect virulence [38].

This is the first report of polymyxin-resistant and XDR* A. baumannii* from Malaysia. The emergence of XDR and polymyxin-resistant* A. baumannii* isolates from the main tertiary hospital in Terengganu, Malaysia, is a cause of concern. Stricter enforcement of antibiotic usage and an antibiotic stewardship program are clearly needed in the hospital to prevent the spread of XDR* A. baumannii*.

## 5. Conclusions

Multidrug resistant* A. baumannii* was observed in 39 of 54 isolates (72.2%) that were obtained from the main tertiary hospital in Terengganu, Malaysia, throughout 2011. High incidence of resistance to carbapenems (about 75%) was noted. Of concern, 14 of the 54 isolates (25.9%) were characterized as extensive drug resistant and were resistant to polymyxin B, considered the drug of “last resort.” Pulsed-field gel electrophoresis indicated that the polymyxin-resistant isolates were genetically diverse. A P102H mutation was found in the* pmrA* gene in all polymyxin-resistant isolates. In addition, novel mutations within the LPS biosynthesis genes* lpxC*,* lpxD,* and* lpsB* were detected in the polymyxin-resistant isolates. Whether these mutations contribute to the development of polymyxin resistance in* A. baumannii* requires further investigations.

## Figures and Tables

**Table 1:** Primers used in this study.

Genes to be amplified	Primer sequences (5′-3′)	PCR conditions	Expected product size (bp)	References
16S rDNA	27F: AGAGTTTGATCMTGGCTCAG 152R: AGGAGGTGWTCCARCC	Initiate—95°C, 3 min; 34 cycles of 95°C, 50 s; 60°C, 50 s; 72°C, 1 min; and final extension at 72°C, 90 s.	1500	[18]
*bla*_ OXA-23_	bla_oxa-23F: GATCGGATTGGAGAACCAGA bla_oxa-23R: ATTTCTGACCGCATTTCCAT	Initiate—95°C, 5 min; 30 cycles of 94°C, 25 s; 2°C, 40 s; 72°C, 50 s and final extension at 72°C, 6 min.	501	[26]
*bla*_ OXA-51_	bla_oxa-51F: TAATGCTTTGATCGGCCTTG bla_oxa-51R: TGGATTGCACTTCATCTTGG	Initiate—95°C, 3 min; 34 cycles of 95°C, 35 s; 57°C, 35 s; 72°C, 45 s; and final extension at 72°C, 90 s.	353	[26]
*bla*_ OXA-24_	bla_oxa-24F: GGTTAGTTGGCCCCCTTAAA bla_oxa-24R: AGTTGAGCGAAAAGGGGATT	Initiate—95°C, 5 min; 30 cycles of 94°C, 25 s; 52°C, 40 s; 72°C, 50 s; and final extension at 72°C, 6 min.	246	[26]
*bla*_ OXA-58_	bla_oxa-58F: AAGTATTGGGGCTTGTGCTG bla_oxa-58R: CCCCTCTGCGCTCTACATAC	Initiate—95°C, 3 min; 30 cycles of 94°C, 1 min; 55°C, 1 min; 72°C; 1 min; and final extension at 72°C, 5 min.	599	[26]
*bla*_ IMP_	blaIMP-F: CTACCGCAGCAGAGTCTTTG blaIMP-R: AACCAGTTTTGCCTTACCAT	Initiate—95°C, 5 min; 34 cycles of 94°C, 25 s; 58°C, 40 s; 72°C, 50 s; and final extension at 72°C, 6 min.	692	[2]
*bla*_ VIM_	blaVIM-F: GTTTGGTCGCATATCGCAAC blaVIM-R: CTACTCAACGACTGAGCCATTTGT	Initiate—95°C, 5 min; 34 cycles of 94°C, 1 min, 58°C, 40 s; 72°C, 50 s; and final extension at 72°C, 6 min.	643	[2]
*pmrC *	pmrC_F: ATGTTTAATCTCATTATAGCCATTTG pmrC_R: TTAGTTTACATGGGCACAAGAGTG	Initiate—95°C, 3 min; 35 cycles of 95°C, 35 s, 56°C, 35 s; 72°C, 45 s; and final extension at 72°C, 90.	1602	This study
*pmrA *	pmrA_F: ATGACAAAAATCTTGATGATTGAA pmrA_R: TTATGATTGCCCCAAACGGTA	Initiate—95°C, 3 min; 35 cycles of 95°C, 35 s; 56°C, 35 s; 72°C, 45 s; and final extension at 72°C, 90 s.	675	This study
*pmrB *	pmrB_F: GACTGATTTGGGGCACCTC pmrB_R: TGTTTCATGTAAATGTAAAACTTTAGG	Initiate—95°C, 3 min; 35 cycles of 95°C, 35 s; 56°C, 35 s; 72°C, 45 s; and final extension at 72°C, 90 s.	1304	This study
*lpxA *	lpxA_F: TGAAGCATTAGCTCAAGTTT lpxA_R: GTCAGCAAATCAATACAAGA	Initiate—95°C, 3 min; 35 cycles of 95°C, 35 s; 56°C, 35 s; 72°C, 45 s; and final extension at 72°C, 90 s.	1179	[30]
*lpxC *	lpxC_F: TGAAGATGACGTTCCTGCAA lpxC_R: TGGTGAAAATCAGGCAATGA	Initiate—95°C, 3 min; 35 cycles of 95°C, 35 s; 55°C, 35 s; 72°C, 45 s; and final extension at 72°C, 90 s.	1164	[30]
*lpxD *	lpxD_F: CAAAGTATGAATACAACTTTTGAG lpxD_R: GTCAATGGCACATCTGCTAAT	Initiate—95°C, 3 min, 35 cycles of 95°C, 35 s; 55°C, 35 s; 72°C for 45 s; and final extension at 72°C, 90 s.	1502	[30]
*lpsB *	lpsB_F: GCCCGAATTCGCTTCGTATCGCACCAACTC lpsB_R: CCCGGATATCTCAATTCAATACACTTTGATATAGCTC	Initiate—95°C, 3 min; 35 cycles of 95°C, 35 s, 58°C, 35 s, 72°C, 45 s and final extension at 72°C, 90 s.	1400	[14]

**Table 2:** Mutational points found in the polymyxin-resistant determinants among the 14 polymyxin-resistant *A. baumannii* isolates as compared to susceptible isolates.

Isolate	Mutations			
	*pmrA *	*lpxD *	*lpxC *	*lpsB *
AC12	P102H	S102T, V141I, and R173G	K141R	H181Y
AC30	P102H	S102T, V141I, and R173G	K141R	H181Y
AC3	P102H	T104K, I178V	S158R	—
AC13	P102H	T121A, N151D, and G169S	S158R	—
AC16	P102H	E50D, V141I	K141R	H181Y
AC17	P102H	E50D, V141I	K141R	H181Y
AC18	P102H	E50D, V141I	K141R	H181Y
AC20	P102H	T15A, T121A, N151D, and G186S	K141R, S158R	H181Y
AC21	P102H	T15A, T121A, N151D, and G186S	K141R, S158R	—
AC22	P102H	E50D, S102T, and I178V	K141R, S158R	H181Y
AC27	P102H	S102T, G169S, and R173G	S158R	H181Y
AC38	P102H	S102T, G169S, I178V, and G186S,	K141R	—
AC51	P102H	E50D, R173G	K141R, S158R	—
AC61	P102H	I178V, S181N	K141R, S158R	—

^*∗*^Note: no mutation was detected in *pmrC*, *pmrB,* and *lpxA*.
